# The role of Gamma Knife radiosurgery in the management of skull base chordoma

**DOI:** 10.3389/fonc.2022.1046238

**Published:** 2023-02-09

**Authors:** Kuanyu Wang, Dezhi Gao, Jian Pan, Enmeng Bao, Shibin Sun

**Affiliations:** ^1^ Gamma Knife Center, Beijing Tiantan Hospital, Capital Medical University, Beijing, China; ^2^ Gamma Knife Center, Beijing Neurosurgical Institute, Capital Medical University, Beijing, China

**Keywords:** Gamma Knife radiosurgery (GKS), chordoma, MDT, multi-procedure radiosurgery, multi-modality imaging

## Abstract

**Objective:**

Chordoma is a slow-growing and locally aggressive cancer, which arises from the remnants of the primitive notochord. The first line treatment for the skull base chordoma is neurosurgery. Gamma Knife radiosurgery (GKS) is often be chosen especially in the setting of residual or recurrent chordomas. The purpose of this study is to evaluate the prognosis of patients with skull base chordoma who underwent GKS.

**Methods:**

The present study was a retrospective analysis of 53 patients with skull base chordomas who underwent GKS. Univariate Cox and Kaplan-Meier survival analysis were performed to analyze the relationship between the tumor control time and the clinical characteristics.

**Results:**

The 1-, 2-, 3-, and 5-year progression free survival (PFS) rates were 87, 71, 51, and 18%, respectively. After performing the univariate analysis, the clinical characteristics were not found to be significantly associated with the time of PFS; however, surgical history, peripheral dose, and tumor volume did have tendencies to predict the prognosis.

**Conclusion:**

GKS provided a safe and relatively effective treatment for residual or recurrent chordomas after surgical resection. A higher tumor control rate depends on two approaches, an appropriate dose of radiation for the tumor and the accurate identification of the tumor margins.

## Introduction

Ribbert first described the term chordoma in 1894, as having a rare incidence rate of about 0.1 per 100,000 individuals (1). Chordoma, which arises from the remnants of the primitive notochord, is characteristically slow-growing and locally aggressive (2), and occurs more frequently in adults than children, with most diagnoses occurring between 40 to 60 years of age (3). Chordomas often occur in the skull base region (35%), the sacrococcygeal region (50%), and the vertebrae region (15%) (3, 4).

According to the World Health Organization (WHO) classification system, chordoma is divided into 3 distinct types: conventional chordoma (chondroid chordoma), dedifferentiated chordoma, and poorly differentiated chordoma (5). Patients with untreated chordomas have poor prognoses, with a mean survival < 1 year (6).

Although there is no doubt that neurosurgical therapy is the first line treatment option for the skull base chordoma, a total resection is often difficult to obtain, due to the invasion of the tumor into skull base and its location being adjacent to numerous important structures, along with a high tumor recurrence rate (7). The chemotherapy and traditional low-dose radiotherapy is not sensitive to this tumor (8). It is difficult to perform a repeat neurosurgical resection, necessitating the utilization of various radiotherapy approaches, including Gamma Knife radiosurgery (GKS), in an effort to control the tumor recurrence (9). GKS has been used for residual or recurrent chordoma as an effective treatment. In this study, we performed a large single-institution retrospective review of the prognosis for the skull base chordoma patients who underwent GKS. The purpose of this study is to define the role of GKS in the treatment of the chordoma patients.

## Materials and methods

### Patient demographics

We identified a total of 53 consecutive patients with chordomas who underwent GKS in Tiantan Hospital Gamma Knife Center between January 2006 and December 2019. The initial treatment for 52 of the patients was microsurgical resection, and the diagnosis of chordoma was confirmed by histopathology prior to the patients undergoing GKS, except for the 53rd patient, who underwent a remedial surgical resection post-GKS. Of the 53 patients, 34 (64%) were male and 19 (36%) were female. The mean age was 45 years (range, 16–71 years). Of the 53 patients, 8 had previously undergone radiotherapy – 5 underwent external beam radiotherapy and 3 underwent stereotactic radiosurgery (2 with GKS and 1 with cyber knife). Adjuvant GKS for the treatment of residual or recurrent chordomas occurred at an average of 11.9 months (range, 1–60 months) after the initial surgical resection. Of the 52 patients who were treated with salvage GKS, 1 patient underwent 8 surgical resections prior to GKS, 12 underwent 2 surgical resections, and 39 underwent 1 surgical resection (**Table 1**). Written informed consent was obtained from all patients involved in the present study.

**Table 1:** The basic information.

	Mean	Range
Gender	Male, 34	
	Female, 19	
Age	45	16–71
Surgery History	None, 1	
	Yes, 53	
Prior Radiotherapy	None, 45	
	Yes, 8	
Postoperative Interval (months)	12	1–60
Tumor Volume (cm^3^)	17.1	1.08–62.6
Peripheral Dose (Gy)	13.5	10.0–16.0
Central Dose (Gy)	29.4	22.0–33.3
Follow-up (months)	53	8–168
EQD2 (Gy)	44.8	26–60.8

### Radiosurgical parameters

In all 53 cases, a Leksell stereotactic frame was fixed to the patient’s skull under local anesthesia. High-resolution contrast-enhanced magnetic resonance imaging (MRI) was performed, utilizing a slice thickness of 2 mm for treatment planning. Radiosurgery was performed using the Leksell Gamma Knife model B (Elekta AB) before 2007, the Leksell Gamma Knife model C (Elekta AB) between January 2007 and October 2011, and the Leksell Gamma Knife Perfexion (Elekta AB) thereafter. Multiple dose planning sessions were carried out using the GammaPlan system (Elekta Instruments).

In the cohort evaluated in the present study, the mean prescribed radiation dose was 13.5 Gy (range, 10–16 Gy), for which the equivalent dose in 2-Gy fractions (EQD2; calculated assuming an α/β = 3 for chordomas) ranged from 26 to 60.8 Gy (mean, 44.8 Gy). The mean isodose line was 45.8% (range, 40–50%); the mean maximum dose was 29.4 Gy (range 22–33.3 Gy); and the mean tumor volume was 17.1 cm^3^ (range, 1.1–62.2 cm^3^). Some patients had complicated radiotherapy histories, so the average total EQD2 for these patients was 60.3 Gy (range, 26–200 Gy) (**Table 1**).

### Follow-up and statistical analysis

All patients were recommended to undergo MRI and clinical evaluations at 6-month intervals during the first year post-GKS, and every 6–12 months thereafter. Tumor recurrence was defined as an increase in the tumor size after GKS, or the development of a new tumor, as seen on follow-up MRI. Tumor recurrences were categorized as local (enlargement of the treated tumor), marginal (new tumor formation out of the prescribed line, but within the resection cavity), and distant (new tumor formation away from the resection cavity). In order to account for the variability between scanners and images, tumor shrinkage or growth was defined as a 25% decrease or increase in volume, respectively. Univariate Cox and Kaplan-Meier survival analysis were performed to analyze the relationship between the tumor control time and the clinical characteristics.

## Results

For the present study, the mean duration of follow-up was 53 months (range 6–115 months). Of the 53 patients included in this study, follow-up MRI demonstrated that only 10 (19%) had good tumor control which remained stable, while 43 (81%) had varying degrees of tumor development, for an overall total tumor control rate of 19%. Of the 43 patients who experienced tumor development, 15 underwent repeat GKS, 1 underwent local radiotherapy, 12 underwent repeat surgical resection, 15 received only clinical observation or ceased follow-up, and 2 died from repeated tumor recurrence. The pattern of recurrence was as follows: local + marginal for 35 patients; distant for 3 patients; and local + marginal + distant for 5 patients. The 1-, 2-, 3-, and 5-year progression free survival (PFS) rates were 87, 71, 51% and 18%, respectively. Complex treatment strategies were adopted for most patients through their last follow-up, undergoing surgical resections as follows: 32 patients underwent a single surgical resection; 14 underwent 2 surgical resections; 5 underwent 3 surgical resections; 1 underwent 4 surgical resections; and 1 underwent 8 surgical resections. The patients underwent radiotherapy treatments as follows: 25 underwent a single radiotherapy treatment; 15 underwent 2 radiotherapy treatments; 7 underwent 3 radiotherapy treatments; 4 underwent 4 radiotherapy treatments; 1 underwent 5 radiotherapy treatments; and 1 underwent 6 radiotherapy treatments.

**Figure 1:**
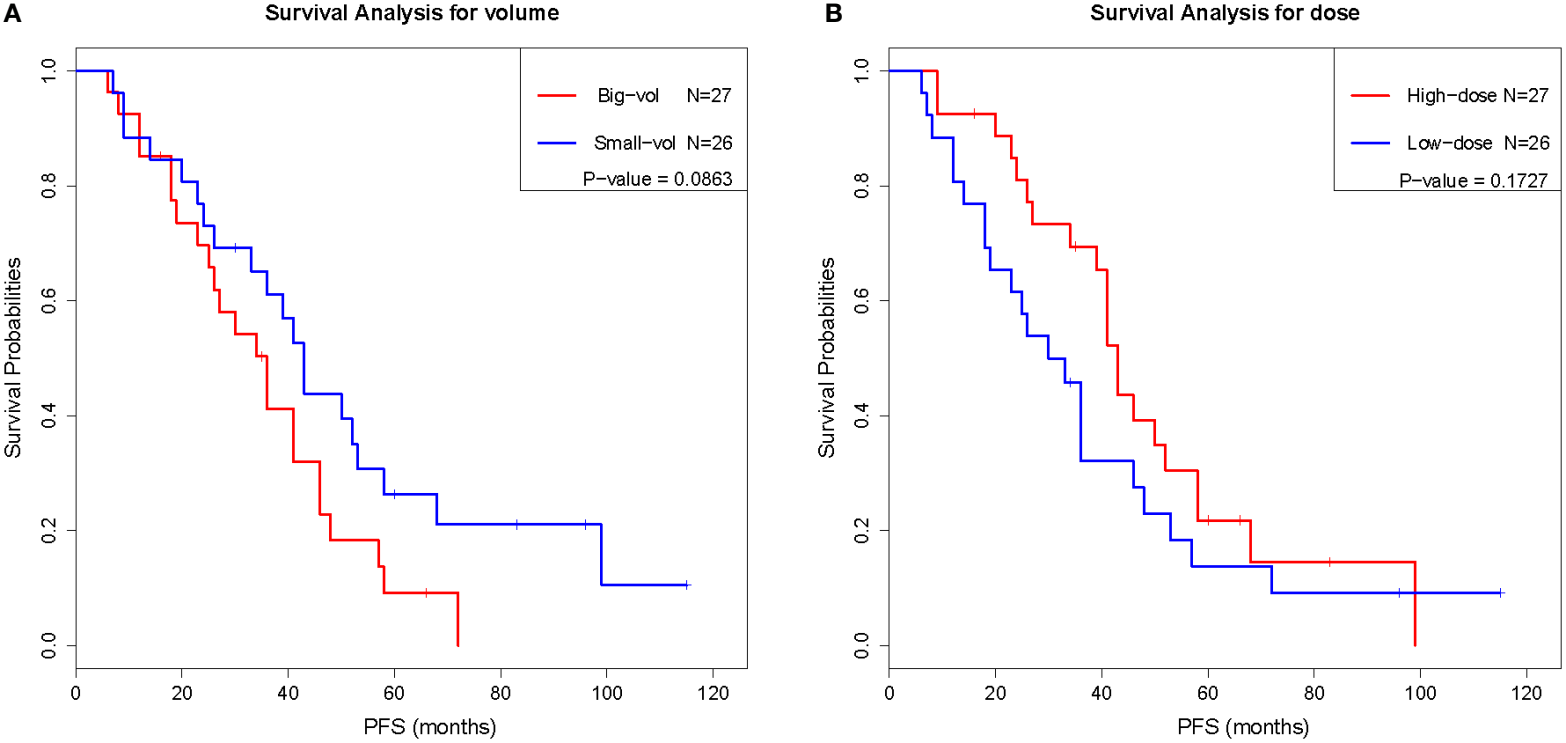
Kaplan-Meier analysis of tumor volume **(A)** and peripheral dose **(B)**.

To evaluate the prognostic value of various factors in patients with chordomas who underwent GKS, the univariate Cox regression analysis was performed. The results of this analysis showed that several factors, including sex, age, surgical history, postoperative interval, number of shots, central dose, peripheral dose, and tumor volume, were not significantly associated with PFS (*P* ≥ 0.05) (**Table 2**). Although there was no significant difference (*P* < 0.05) between these factors and PFS, the trend was existing, as the surgical history, peripheral dose, and tumor volume tended to predict the prognosis. Similar results were also obtained by Kaplan-Meier analysis (**Figure 1**), the results of which implied that PFS might be related to the surgical history, peripheral dose, and tumor volume in chordoma patients.

**Table 2:** Univariate analysis.

	HR	95.0% CI	*P*-value
Sex	1.87	0.96–3.65	0.07
Age	1.00	0.98–1.03	0.77
Surgical History	1.17	0.94–1.45	0.15
Postoperative Interval	0.99	0.96–1.02	0.54
Central Dose	0.95	0.85–1.05	0.29
Peripheral Dose	0.86	0.69–1.06	0.16
Tumor Volume	1.01	0.99–1.03	0.19
EQD2	0.97	0.94–1.00	0.11

## Discussion

Chordomas are often considered to be low-grade malignancies; however, the tumor cells frequently show locally aggressive growth, leading to a higher recurrence rate after the initial neurosurgical resection. The standard treatment for clival chordomas is complete or subtotal neurosurgical resection, while radiotherapy treatments are necessary for the residual or recurrent tumors after surgical management to improve local tumor control (10). The results of retrospective studies from multiple institutions have shown that GKS plays an important role in the treatment of residual or recurrent chordomas.

The first clinical report of GKS for chordoma was published in 1991 by Kondziolka from the University of Pittsburgh Medical Center, which revealed the confirmed efficacy of GKS with 20 Gy to the tumor margin for four patients. Subsequently Muthukumar et al. (11) also from the Pittsburgh group reported 9 patients with chordoma treated with GKS in 1998. A multicenter study investigated by Kano showed that the skull base chordomas patients who underwent GKS had a 5-year local control rate of 66% and a 5-year survival of 80%. The median marginal doses of 12.7–20 Gy were used in these studies of GKS for chordoma from **Table 3**. The clinical data from this table showed that the 5-year local control rate of chordoma after Gamma Knife treatment was 21.4%–76%, and the 5-year survival was 67-100 (1, 7, 11–20) (**Table 3**). The tumor control rates from these clinical investigations varied widely, which demonstrated the radio-resistant characteristic of chordomas.

**Table 3:** Summary table of GKS studies for chordoma.

Author	Year	No.	Vol. (cm3)	Median Prescription Dose (Gy)	5-years Local Control (%)	5-years Survival (%)	Follow-Up (months)
Muthukumar et al.	1998	9	NA	18	66	NA	48
Krishnan et al.	2005	25	14.4	15	32	90	58
Hasegawa et al.	2007	27	19.7	14	76	80	59
Martin et al.	2007	18	9.8	16	63	63	NA
Liu et al.	2008	31	11.4	12.7	21.4	75.8	30.2
Dassoulas et al.	2009	15	5.8	15.3	50	NA	88
Ito et al.	2009	19	3.3	17.8	47.9	100	87
Koga et al.	2010	14	NA	15	43	NA	65
Kano et al.	2011	71	7.1	15	66	80.2	60
Mori et al.	2014	7	5.1	16.9	67	100	NA
Kim et al.	2014	5	10.7	20	35	73	53
Hafez et al.	2019	12	7	14.7	25	NA	45
Cahill et al.	2021	15	10	20	67	67	84

For the patients who underwent GKS at our center, the 3- and 5-year local control rates were 51 and 18%, respectively. As early as 2008, Liu et al. (21) had completed a retrospective study of 31 patients with skull base chordoma who underwent radiosurgery in our center. The results of that study showed that the 3- and 5-year local control rates were 64.2 and 21.4%, respectively. The mean follow-up time after the radiosurgery treatment was 30.2 months (range, 6–102 months) (21). In the present study, the mean follow-up was extended to 38.9 months (range, 6–115 months); however, there was no significant improvement in the tumor control rate between the two clinical investigations performed at our center. It is believed that the relatively low control effect may be related to the relatively large postoperative residual tumor volume, the identification of the target area, and the relatively low peripheral radiation dose. From our experience, the ideal approach to improve tumor control of chordomas post-GKS depended on three aspects: how to appropriately increase the prescribed radiation dose for each tumor; how to strengthen the multi-disciplinary treatment (MDT) for each patient; and how to determine the real biological margins of each tumor.

Although the differences were not significant, the patient’s surgical history, tumor volume, and peripheral dose had some correlation with the tumor control effect. Perfect total surgical resection and an appropriate increase in the peripheral radiation dose may improve the post-GSK tumor control in patients with chordoma. Several studies have reported that there was a correlation between the local control rate and the radiation dose, such as Koga et al. (18), who reported a significant difference between patients treated with GKS at different doses (18 vs. 12 Gy). The median marginal dose used in the study of Cahill et al. (1) was 20 Gy which is higher than many other studies, the tumor control rates at 5 and 10 years in the chordoma group were 67% and 49%, and the 5- and 10-year overall survival rates were 67% and 53% respectively. In the clinical practice, 20 Gy of prescription margin dose per fraction was difficult to be administered because of the adjacent critical structure. A prescription marginal dose of at least 16 Gy per fraction should be recommended to improve tumor control effect. Multi-procedure radiosurgery, including multi-stage radiosurgery and fractionated radiosurgery, could be performed to achieve the boost of irradiation dose.

To improve tumor control rates in patients with chordoma, it is necessary to introduce the concept of MDT to the diagnosis and treatment process. Neurosurgeons need to remove as much of the tumors as possible, on the premise of preserving nerve function and reducing the residual tumor volume. Radiologists and radiosurgeons should detect any residual tumor or tumor recurrence and perform a radiosurgical treatment as soon as possible. They also should pay attention to the location of the target area and appropriately increase the radiation dose, as necessary. We have recommended that the ideal window of opportunity for radiotherapy intervention when treating residual chordomas is approximately 3 months after the initial surgical resection.

The precise definition of the tumor target volume is crucial to the radiosurgery process, and for this it is necessary to determine which imaging techniques should be utilized for the identification of the real target area. The CT, MRI and PET characteristic features of chordomas have been revealed. CT displays osteolysis and a soft tissue component in almost all cases and frequently contain mineralized matrix or sequestered bone. The T1 sequence of MRI displays isointense signal with heterogeneous contrast enhancement. The T2 sequence displays a high signal intensity with heterogeneous hypointensity associated with mucous, hemorrhage, and calcification. Small foci of internal increased T1 and predominant T2 hyperintensity may also appear on MRIs of chordomas (22). PET generally displays heterogeneous ^18^F-FDG activity with moderate but variable avidity. From our experience, the T2 sequence is of equal importance to the T1 contrast sequence in the MRI localization of the chordoma to outline the real tumor margin for GKS. Recent studies have demonstrated the uptake of multiple positron-emission tomography (PET) tracers in chordomas, which may have an important role in the diagnosis, follow-up, and personalized treatment of chordomas (23). We consider that multi-modality imaging localization fused with MRI, CT, and PET/MR or PET/CT will be utilized in the future for the identification of chordoma tumor margins to achieve higher local tumor control rate.

Additionally, the results of some recent studies have shown that certain adjuvant treatments also play an important role in improving disease control rates in patients with chordomas. A study published in 2021 by DeMaria et al. (2) showed that a novel yeast-brachyury vaccine could be used to treat patients with unresectable chordoma. The results, however, showed that this vaccine did not significantly improve the efficacy of radiotherapy in patients with chordoma (2). Another study, published in 2018 by Gregory et al. described the use of nilotinib, a kind of platelet-derived growth factor receptor (PDGFR) inhibitor, in addition to radiation in the treatment of high-risk chordomas. The results of their study showed that the median PFS was 58.15 months, with a 2-year overall survival rate was 95% (24). Studies such as these provide a research direction for the utilization of MDT in the treatment of chordomas.

This research was a retrospective study and the limitation of our study was that the number of cases was relatively small. The reason was related to the rare incidence of this disease. The univariate Cox analysis had no statistical significance might be related with the small sample size. In the future, we will further expand the sample size through multi-center cooperation.

## Conclusions

GKS provides a safe and relatively effective management for residual or recurrent chordomas after the initial surgical resection. To achieve higher local tumor control of GKS for chordomas, three approaches can be recommended: Multi-procedure radiosurgery to increase the prescription margin dose, MDT management for the patients with chordomas, and Multi-modality imaging fusion to obtain the accurate identification of the tumor margins.

## Data availability statement

The raw data supporting the conclusions of this article will be made available by the authors, without undue reservation.

## Ethics statement

The study was reviewed and approved by the Ethics Review board of Beijing Tiantan Hospital and the written informed consent to participate in this study was obtained.

## Author contributions

SS and KW designed the study, drafted the manuscript and revised the manuscript. DG, JP and EB contributed to the collection of the data. All authors read and approved the final manuscript.
