# Supplementary material for: The role of Gamma Knife radiosurgery in the management of skull base chordoma
Source: Front Oncol. 2023 Feb 9;12:1046238. doi: 10.3389/fonc.2022.1046238 (PMC9947462; doi:10.3389/fonc.2022.1046238)
Supplement: Supplementary file 1 [file Table_1.docx]

| The salvage treatment for the patients failed GKS | |
| --- | --- |
| Treatment | No. |
| Repeat GKS | 15 |
| Local Radiotherapy | 1 |
| Repeat Surgical Resection | 12 |
| Clinical Observation or Loss of Follow-up | 15 |
